# Idiopathic Short Stature in the Genomic Era: Integrating Auxology, Endocrinology, and Emerging Genetic Insights

**DOI:** 10.3390/children12070855

**Published:** 2025-06-27

**Authors:** Roberto Paparella, Arianna Bei, Irene Bernabei, Francesca Tarani, Marcello Niceta, Ida Pucarelli, Luigi Tarani

**Affiliations:** 1 Department of Maternal Infantile and Urological Sciences, Sapienza University of Rome, 00161 Rome, Italy; arianna.bei@uniroma1.it (A.B.); irene.bernabei@uniroma1.it (I.B.); francesca.tarani@uniroma1.it (F.T.); marcello.niceta@opbg.net (M.N.); i.pucarelli@policlinicoumberto1.it (I.P.); luigi.tarani@uniroma1.it (L.T.); 2 Department of Molecular Genetics and Functional Genomics, Ospedale Pediatrico Bambino Gesù, IRCCS, 00146 Rome, Italy

**Keywords:** idiopathic short stature, pediatric endocrinology, auxology, growth hormone, genetic testing

## Abstract

Idiopathic short stature (ISS) represents one of the most frequent yet enigmatic conditions in pediatric endocrinology. Traditionally defined by auxological parameters in the absence of identifiable causes, ISS has long served as a diagnosis of exclusion. However, with the advent of next-generation sequencing, our understanding of the etiological landscape has significantly evolved. Recent studies have revealed that many children previously labeled as idiopathic actually harbor monogenic variants in genes related to the growth hormone–insulin-like growth factor axis, extracellular matrix components, or growth plate signaling pathways. This review integrates auxological assessment with current knowledge on molecular diagnostics to propose a more accurate and individualized approach to short stature. We examine emerging genotype–phenotype correlations, criteria for selecting candidates for genetic testing, and implications for recombinant human growth hormone therapy. Additionally, we advocate for a shift in clinical mindset: from a descriptive to a biologically grounded framework. ISS should be regarded as a transitional label pending further endocrine and genetic clarification. Recognizing this paradigm shift will improve diagnostic accuracy, personalize treatment strategies, and ultimately enhance care for children with growth failure in the genomic era.

## 1. Introduction

Short stature is a common reason for pediatric endocrinology referral, affecting approximately 2–3% of children worldwide [1]. Among these, a significant proportion fails to meet the criteria for known pathological conditions such as growth hormone deficiency (GHD), chronic systemic disease, skeletal dysplasias, or genetic syndromes [2]. These children are often categorized under the umbrella term “idiopathic short stature” (ISS) [3], defined as a height below −2 standard deviation scores (SDS) for age and sex, with no identifiable cause after standard evaluation [4].

The concept of ISS originated in the 1990s as a diagnostic and therapeutic construct, particularly in the context of recombinant human growth hormone (rhGH) treatment authorization [5,6]. However, ISS is inherently heterogeneous and includes various subpopulations: familial short stature (FSS), constitutional delay of growth and puberty (CDGP), and children with subtle or unidentified syndromic features. Despite decades of research, ISS remains a diagnosis of exclusion, often used to label children for whom the biological cause of short stature is not apparent using conventional methods [7].

Recent advances in genetic testing—especially next-generation sequencing (NGS), whole exome sequencing (WES), and targeted gene panels—have revealed that a substantial proportion of children previously classified as having ISS actually harbor monogenic causes of growth failure [8,9,10]. Genes involved in the growth hormone (GH)–insulin-like growth factor-1 (IGF-1) axis, extracellular matrix composition, paracrine signaling at the growth plate, and transcriptional regulation have been implicated [11,12,13].

These discoveries challenge the classical dichotomy between “idiopathic” and “pathological” short stature. They also call for a revision of diagnostic algorithms and therapeutic strategies to reflect the new genomic landscape [14]. This narrative review aims to (1) examine the historical and auxological context of ISS, (2) explore recent genetic discoveries, (3) propose a pathway-based framework for understanding short stature, and (4) discuss the clinical implications of integrating genetics into pediatric growth assessment.

## 2. Auxology and the Historical Concept of ISS

Auxological assessment remains the cornerstone of short stature evaluation. Precise longitudinal measurements of height, growth velocity, and pubertal timing, combined with evaluation of target height, allow clinicians to detect abnormal growth trajectories early [15,16]. However, in the genomic era, auxology is no longer merely descriptive: it serves as a phenotypic lens through which complex genetic underpinnings can be suspected, stratified, and interpreted.

Within this framework, patients with ISS often present with mild but persistent deviations in height (−2 to −3 SDS) without dysmorphic features or systemic disease [17]. The heterogeneity within ISS is significant. Classic subtypes include the following:

- FSS: children grow along a percentile track consistent with family history and bone age is typically normal [18].
- CDGP: characterized by delayed bone age, slow growth in childhood, and late pubertal onset, often with normal final adult height [19].
- Non-familial ISS: children without obvious familial traits or delayed puberty, sometimes with subtle dysmorphic features or unexplained growth failure.

While auxological red flags—such as disproportionate growth, decelerating velocity, or discrepancy from parental height—traditionally raise suspicion for syndromic or endocrine causes, they are also increasingly recognized in monogenic forms of ISS, including those involving epigenetic regulators and cartilage matrix genes.

Growth hormone stimulation tests have been used to distinguish GHD from ISS. However, these tests are hampered by poor reproducibility, variable cutoff thresholds, and limited predictive value for treatment response [20]. Furthermore, many children with ISS exhibit normal serum IGF-1 and IGF binding protein-3 (IGFBP-3) levels, further complicating the diagnostic picture.

The introduction of rhGH treatment for ISS in the early 2000s was based on evidence showing modest improvements in final adult height—typically around 3–7 cm depending on timing, dose, and duration [21]. Yet, this approach remained empirical, often without clarity regarding which children would benefit most. The result has been a clinical impasse: children with short stature but no clear diagnosis, uncertain prognosis, and variable treatment response. In this context, ISS has increasingly been criticized as a “wastebasket diagnosis,” reflecting diagnostic uncertainty more than biological homogeneity [22]. The genomic era offers tools to overcome this limitation. Emerging evidence supports the integration of machine learning-based growth prediction models, which can identify subtle deviations from expected trajectories not easily detected by classical charts [23]. However, these models are not yet widely validated across ethnicities and clinical settings and often fail to account for the molecular heterogeneity behind short stature.

In this context, auxology plays a dual role: it remains a key clinical entry point but also acts as a biological phenotype that reflects the functional impact of genetic variation on the growth plate. Certain auxological patterns—such as severe short stature despite normal IGF-1, delayed bone age with mild dysmorphisms, or reduced sitting height/height ratio—should prompt early consideration of genetic testing, even in the absence of syndromic features [24].

Ultimately, a modern auxological evaluation must be dynamic, longitudinal, and genomically informed. Rather than anchoring the diagnosis in static centile charts, clinicians are encouraged to link growth data with molecular findings, enabling more precise phenotyping and interpretation of genetic variants of uncertain significance (VUS) within growth-related pathways [25].

## 3. The GH–IGF-1 Axis and Beyond: A Pathway-Based Framework

The GH–IGF-1 axis is central to postnatal linear growth. GH secreted by the pituitary stimulates hepatic production of IGF-1, which acts through endocrine and paracrine pathways to promote chondrocyte proliferation and hypertrophy at the growth plate [26]. Classic disorders of this axis—such as GH deficiency, GH receptor defects, and *IGF-1* gene mutations—are well-established causes of short stature [27].

However, many children with ISS have normal GH secretion and IGF-1 bioavailability, suggesting alternative mechanisms. Genetic investigations have identified several categories of genes implicated in ISS Table 1.

Linear bone growth occurs at the epiphyseal growth plate, a dynamic structure composed of organized columns of chondrocytes embedded within a specialized extracellular matrix (ECM). The interplay between proliferating columnar chondrocytes, ECM components, and tightly regulated paracrine signaling governs the progression from proliferation to hypertrophy and eventual ossification. The C-type natriuretic peptide (CNP)–natriuretic peptide receptor (NPR)-2–cyclic guanosine monophosphate (cGMP) pathway promotes chondrocyte hypertrophy and ECM expansion, antagonizing the growth-inhibitory effects of fibroblast growth factor receptor 3 (FGFR3) signaling, which acts through mitogen-activated protein kinase (MAPK) to limit proliferation and induce premature maturation [28]. Indian hedgehog, produced by prehypertrophic chondrocytes, mediates a feedback loop with parathyroid hormone-related protein to maintain proliferative potential and delay hypertrophy [29].

**Figure 1:**
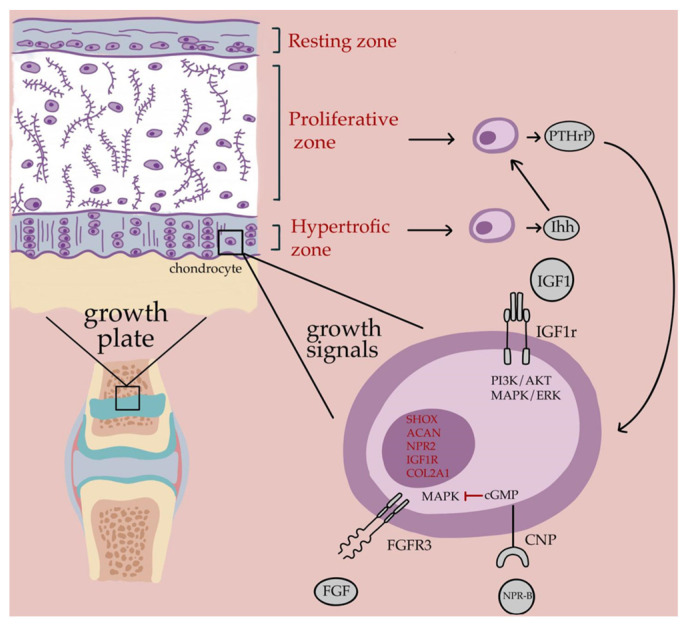
Schematic representation of the growth plate and key molecular pathways in linear bone growth. The growth plate is organized into resting, proliferative, and hypertrophic zones. Chondrocyte proliferation and differentiation are regulated by endocrine, paracrine, and autocrine signals. The PTHrP–Ihh loop maintains proliferation; IGF-1 promotes maturation via IGF1R-mediated PI3K/AKT and MAPK/ERK pathways; CNP–NPR2–cGMP signaling enhances hypertrophy and counteracts FGFR3-mediated inhibition. Genes commonly associated with idiopathic short stature, including *SHOX*, *ACAN*, *NPR2*, *IGF1R*, and *COL2A1* (in red), modulate these pathways and influence growth outcomes.

The GH–IGF-1 axis exerts systemic and local effects, with IGF-1 acting via IGF1 receptor (IGF1R) to stimulate chondrocyte proliferation, hypertrophy, and ECM synthesis [30]. Structural ECM proteins such as aggrecan (*ACAN*), essential for cartilage hydration and compressive resistance, are directly regulated by IGF-1 signaling and are critical for the maintenance of normal growth plate architecture [31,32]. *SHOX*, a transcription factor expressed in hypertrophic and perichondrial regions, modulates growth plate development through downstream targets such as FGFR3 and NPPB, and haploinsufficiency is associated with disrupted columnar organization and reduced chondrogenesis [33]. Similarly, *NPR2*, encoding the CNP receptor, is essential for normal endochondral ossification; loss-of-function mutations in *NPR2* are associated with severe short stature, while activating mutations result in tall stature syndromes [34]. Together, these pathways integrate hormonal, paracrine, and structural cues to orchestrate the finely tuned process of longitudinal bone growth Figure 1.

**Table 1:** Selected genes associated with ISS: functions, phenotypes, and clinical notes.

Gene	Function	Phenotype	Comments
*SHOX* [35]	Homeobox transcription factor involved in growth plate regulation	Mesomelic short stature, Madelung deformity, reduced arm span; more severe in females	Common in ISS; test via MLPA or CGH; rhGH therapy often beneficial
*ACAN* [32]	Key structural proteoglycan in cartilage matrix (aggrecan)	Proportionate short stature, advanced bone age, early-onset osteoarthritis, midface hypoplasia	Autosomal dominant; GH response variable; radiography may aid diagnosis
*NPR2* [36]	Receptor for CNP in growth plate chondrocytes	Mild to moderate proportionate short stature, sometimes with advanced bone age	May benefit from CNP analogs; consider in familial ISS
*IGF1R* [37]	Receptor for IGF-1, mediating growth-promoting signals	Intrauterine and postnatal growth restriction, microcephaly, variable intellectual outcome	Variable response to IGF-1 therapy; heterozygous deletions common
*GHR* [38]	Receptor for growth hormone, mediating IGF-1 production	GHI: short stature with high GH and low IGF-1 levels; variable skeletal features	Check in children with GHI pattern; biallelic more severe
*STAT5B* [39]	Transducer in GH signaling pathway via JAK-STAT mechanism	Severe postnatal growth failure with immune dysfunction (recurrent infections, eczema)	Consider in syndromic short stature with eczema or recurrent infections
*COL2A1* [40]	Collagen type II involved in cartilage and skeletal development	Short trunk, scoliosis, early-onset osteoarthritis, possible hearing loss or eye anomalies	Mild forms may resemble ISS; radiology can aid differentiation

Abbreviations: CGH, comparative genomic hybridization; CNP, C-type natriuretic peptide; GH, growth hormone; GHI, growth hormone insensitivity; IGF-1, insulin-like growth factor 1; ISS, idiopathic short stature; JAK, Janus kinase; MLPA, multiplex ligation-dependent probe amplification; rhGH, recombinant human growth hormone; STAT, signal transducer and activator of transcription.

### 3.1. Partial GH Insensitivity

GH insensitivity (GHI) refers to a heterogeneous group of genetic disorders in which impaired GH signaling leads to growth failure despite normal or elevated circulating GH levels [41]. Classical GHI, such as Laron syndrome, results from biallelic mutations in the GH receptor (*GHR*) gene and is characterized by severe postnatal growth retardation, midfacial hypoplasia, delayed puberty, and extremely low serum levels of IGF-I, IGFBP-3, and acid-labile subunit that fail to respond to GH stimulation [42,43]. Similar biochemical and growth profiles are observed in patients with biallelic signal transducer and activator of transcription 5B (*STAT5B*) mutations, though these cases are often complicated by immune dysfunction and elevated prolactin levels [39]. Mutations in *GHR*, *STAT5B*, and *IGFALS* can cause varying degrees of GHI [44]. For example, heterozygous *GHR* mutations may result in a mild phenotype with normal GH and IGF-1 levels, mimicking ISS [26,38].

From a therapeutic perspective, recombinant IGF-I (rhIGF-I) is the primary treatment for patients with classical GHI due to *GHR* defects and is approved for use in Laron syndrome. Treatment typically results in improved height velocity during early years, although the impact on adult height remains modest [45]. The efficacy of rhIGF-I in other forms of GHI is less well-established. In patients with heterozygous IGF1 mutations, preliminary data suggests a potential benefit from rhGH therapy, likely due to residual receptor function and partial downstream signaling capacity [46,47].

Noonan syndrome represents a paradigmatic example of partial GHI due to hyperactivation of the RAS/MAPK pathway, which impairs JAK2–STAT5b signaling and reduces IGF-1 synthesis. Noonan syndrome is a multisystem genetic disorder characterized by short stature, distinctive facial features, congenital heart defects, and variable cognitive deficits. Although it is not considered under the ISS category due to its syndromic presentation, it provides a relevant model of partial GHI with post-receptor signaling defects. Although GH stimulation tests typically show normal or elevated GH secretion, many patients display low-normal IGF-1 and acid-labile subunit protein levels, and subnormal IGF-1 generation in response to exogenous GH administration, reflecting a post-receptor defect. Clinically, rhGH therapy is FDA-approved for Noonan syndrome and produces moderate but clinically significant gains in height SDS (~1.1–1.5 SDS), particularly when initiated early and continued for several years [48,49]. In 3M syndrome, GH treatment shows variable efficacy, and in vitro data suggest combined insensitivity to GH and IGF-I, which may explain the limited clinical benefit [50,51].

Ultimately, the choice of therapy depends on the underlying molecular defect, severity of IGF-I deficiency, and functional integrity of downstream signaling. A personalized treatment approach based on genetic diagnosis is essential for optimizing growth outcomes and avoiding ineffective or unnecessary interventions.

### 3.2. IGF-1 Signaling and Receptor Abnormalities

The IGF1R plays a key role in mediating the growth-promoting effects of IGF-1 at the cellular level, particularly in postnatal chondrocyte proliferation and survival [52]. Variants in *IGF1* and *IGF1R* can impair downstream signaling, leading to postnatal growth failure. *IGF1R* haploinsufficiency is associated with intrauterine growth retardation, microcephaly, and ISS-like phenotypes [37]. Several mutations have been identified, on contrary homozygous loss of IGF1R function activity is likely incompatible with survival [53,54].

Clinical studies have shown that children with *IGF1R* mutations may exhibit reduced responsiveness to rhGH treatment, particularly in the first year of therapy. Biochemically, they often display elevated IGF-1 and IGFBP-3 levels, suggestive of partial IGF-1 resistance. Despite this attenuated response, a subset of these patients achieves meaningful catch-up growth over time. Potential explanations for variable therapeutic outcomes include residual receptor function in missense mutations, direct GH effects on the growth plate, and supraphysiological IGF-1 levels induced by rhGH that may partially bypass receptor-level defects [17,53].

Importantly, the presence of an *IGF1R* mutation does not preclude rhGH treatment but highlights the need for individualized therapeutic planning. Regular monitoring of growth velocity, biochemical markers, and metabolic parameters is recommended to optimize outcomes and manage expectations in this genetically defined subgroup [53].

### 3.3. Growth Plate Matrix and Extracellular Structure

The *ACAN* gene, encoding aggrecan, is a key component of the growth plate extracellular matrix. The ECM of the growth plate is essential for proper endochondral ossification and longitudinal bone growth [55]. Among the genes involved, *ACAN* has emerged as a notable contributor to ISS. Heterozygous mutations cause short stature, advanced bone age, and early-onset osteoarthritis [32]. Other implicated genes include *COL2A1*, *MATN3*, and *COMP*.

Recent studies have expanded our understanding of the role of *ACAN* beyond structural integrity. Functional analyses in human cohorts and chondrocyte models have demonstrated that aggrecan is involved in modulating GH signaling. Specifically, the downregulation of *ACAN* expression leads to reduced chondrocyte proliferation and decreased activation of key GH-responsive mediators such as JAK2, STAT1, STAT5B, and IGF-1, despite normal GHR expression. This suggests that *ACAN* plays an active role in transducing GH signals within the growth plate, linking ECM composition to endocrine responsiveness [56,57].

Clinically, GH treatment in patients with *ACAN* mutations has yielded variable outcomes. The heterogeneity in phenotype—even among individuals with the same mutation highlights the importance of comprehensive phenotypic and molecular evaluation.

### 3.4. Paracrine Signaling Pathways

The *NPR2* gene encodes the NPR-B, which mediates CNP signaling in growth plate chondrocytes. Upon ligand binding, NPR-B stimulates intracellular cGMP production, enhancing chondrocyte hypertrophy, matrix production, and longitudinal bone growth [58]. Loss-of-function variants result in reduced chondrocyte hypertrophy and short stature [36].

Biallelic mutations in *NPR2* cause acromesomelic dysplasia, a severe, disproportionate skeletal dysplasia [59]. In contrast, heterozygous variants lead to milder, often proportionate short stature, and are frequently misclassified as ISS or FSS. Subtle skeletal features, such as mesomelic limb shortening or brachydactyly, may be present but are not universally observed [34,36].

Although biallelic mutations in the *NPPC* gene, encoding CNP, have been associated with severe short stature phenotypes, current evidence does not support a clear association between heterozygous *NPPC* variants and ISS. Nevertheless, heterozygous *NPPC* mutations have recently been identified in patients with proportionate short stature and small hands [60]. Given its regulatory role in the same pathway as *NPR2*, *NPPC* is emerging as a gene of interest in growth biology and may warrant future investigation as genomic data accumulates.

Recent data indicate that *NPR2* variants account for up to 6% of cases of FSS [61,62,63]. Affected children generally respond favorably to rhGH therapy, with gains in height SDS ranging from 1.2 to 1.8 over five years and significant increases in growth velocity during the first year of treatment [62]. This response profile is comparable to that seen in other growth plate disorders such as short stature homeobox-containing gene (*SHOX*) deficiency, for which rhGH therapy efficacy has already been well-established [64,65].

Given its diagnostic yield and therapeutic relevance, *NPR2* should be included in targeted NGS panels for children with unexplained short stature, particularly when there is family history. Early genetic diagnosis can facilitate timely initiation of GH therapy and inform prognosis, underscoring the clinical utility of integrating paracrine signaling pathway genes into the modern diagnostic framework for ISS.

### 3.5. Transcription Factors and Chromatin Regulation

Mutations in genes such as *HMGA2*, *EZH2*, and *NFIX* affect transcriptional regulation of growth-related genes and may present with isolated short stature or syndromic features [66]. Transcription factors and chromatin regulators play pivotal roles in orchestrating the gene expression networks that govern chondrocyte proliferation, differentiation, and endochondral ossification at the growth plate. Mutations in genes encoding these factors can disrupt tightly regulated developmental processes and manifest as idiopathic or syndromic short stature [67].

One of the most well-characterized transcription factors in growth regulation is high-mobility group-A2 (HMGA2), a high-mobility group protein that modulates chromatin structure and transcriptional activity during embryonic and postnatal growth. *HMGA2* variants have been associated with Silver-Russell syndrome-like phenotypes and isolated familial short stature [68]. Its role in mesenchymal cell proliferation and growth plate maturation is supported by both animal models and human data. Loss-of-function mutations in *HMGA2* result in decreased cell proliferation and delayed ossification, reinforcing its importance in linear growth regulation [69].

Another relevant gene is *EZH2*, which encodes a core component of the Polycomb Repressive Complex 2, responsible for the trimethylation of lysine 27 on histone H3 protein subunit, a key epigenetic mark of gene silencing [70]. Heterozygous pathogenic variants in *EZH2* are classically associated with Weaver syndrome, characterized by tall stature and advanced bone age [71]. However, loss-of-function or regulatory variants may lead to growth attenuation and syndromic short stature with variable expressivity. *EZH2* activity modulates growth by regulating downstream targets such as the IGF pathway genes, underscoring the relevance of epigenetic repression in chondrocyte maturation [72].

*NFIX* gene, encoding another transcription factor, regulates mesenchymal stem cell differentiation and skeletal patterning. Germline mutations in *NFIX* can cause either Sotos-like overgrowth (Marshall–Smith syndrome) [73] or undergrowth syndromes depending on the nature of the variant and its effect on transcriptional output. In some patients, *NFIX* variants have been identified in cases of unexplained short stature, highlighting its dosage-sensitive role in skeletal development [74].

Emerging data also point to additional transcriptional regulators such as *SOX11*, *CREBBP*, and *KMT2D*, more commonly associated with neurodevelopmental disorders, but occasionally presenting with growth delay as a leading or isolated feature [75,76,77,78]. These findings support the view that disruptions in chromatin accessibility and transcriptional fine-tuning—whether through transcription factors, histone-modifying enzymes, or chromatin remodelers—can influence stature even in the absence of overt syndromic features [79,80].

Importantly, the diagnostic yield for mutations in transcriptional and epigenetic regulators may increase as exome and genome sequencing are extended to cohorts of children with unexplained growth failure. Their identification offers novel insight into the regulation of growth beyond classical endocrine and paracrine axes and may inform targeted therapies in the future, especially as epigenetic drugs become more refined [81,82,83].

## 4. Genetic Testing in Children with ISS: When and How?

The introduction of NGS has revolutionized our ability to identify monogenic causes of growth disorders. The diagnostic yield varies based on patient selection and testing modality. The prevalence of *SHOX* mutations in ISS generally ranges from about 2% to 15%, with most studies reporting rates between 2% and 7%, but some populations and meta-analyses report higher rates up to 15% [35,84,85,86,87]. Mutations in *ACAN* or *NPR2* are found in about 2–6% [9,88]. According to a recent systematic review and meta-analysis, the diagnostic yield of exome sequencing and chromosomal microarray analysis (CMA) for short stature cohorts is approximately 25–30% and 10–15%, respectively [89].

Genetic testing should be considered in children with [90].

- Height below −3 SDS, with increasing probability to find underlining genetic causes for more severe form of ISS;
- Disproportionate stature;
- Family history of short stature;
- Advanced bone age or skeletal anomalies;
- Poor response to rhGH despite good adherence;
- Presence of additional features (e.g., joint hypermobility, dysmorphisms, developmental delay).

Initial testing strategies may include multiplex ligation-dependent probe amplification (MLPA) or array-comparative genomic hybridization (CGH) to detect *SHOX* deletions. Targeted NGS panels typically include genes involved in the GH–IGF1 axis, extracellular matrix components, and paracrine signaling pathways. In complex or syndromic cases, WES or whole genome sequencing (WGS) may be warranted.

While NGS technologies have revolutionized the diagnostic landscape, their cost and limited availability in low- and middle-income countries remain major barriers to equitable implementation. Infrastructure constraints, lack of trained personnel, and limited insurance coverage further exacerbate disparities. Prioritization strategies—such as using phenotype-driven gene panels and international telemedicine collaborations—may help bridge this gap and improve global access to genetic testing in pediatric endocrinology [91,92].

Variant interpretation must follow ACMG guidelines and involve multidisciplinary review. A major challenge is the classification of VUS, which may not be immediately actionable but can guide future research or family testing [93]. VUS are a frequent outcome of NGS in children with ISS. In the recent study by Cavarzere et al. [90], VUS were identified in over 70% of the cohort, and more than one was found in approximately 61% of these patients, suggesting a potential cumulative or synergistic effect on growth impairment. Although, by definition, VUS lack sufficient evidence for definitive pathogenic or benign classification, their possible role in the etiology of short stature should not be underestimated. Multiple VUS occurring within the same individual may contribute additively to phenotype severity, and in some cases, may co-occur with known pathogenic variants, further complicating clinical interpretation. Functional studies and segregation analyses are essential to reclassify VUS, but such resources are rarely available in routine clinical practice. Bioinformatic predictors, genotype-phenotype correlations, and familial data may assist in interpreting their potential relevance. Importantly, the presence of VUS should prompt careful longitudinal follow-up, particularly when combined with atypical growth patterns or mild dysmorphic features. The recognition of VUS as possible genetic modifiers underscores the complexity of ISS and supports the integration of genetic findings into a broader clinical framework rather than a dichotomous pathogenic/non-pathogenic model. Beyond clinical interpretation, ethical issues surrounding genetic testing in children with ISS warrant careful consideration. Incidental findings, such as predispositions to cancer or neuropsychiatric conditions, pose complex challenges for disclosure and family communication. In pediatric contexts, where informed consent must be revisited over time, genetic data storage and secondary use must be governed by transparent policies and ethical oversight [94,95]. Clinicians should be supported by structured guidelines and ethical counseling services to navigate these issues responsibly. Genetic counseling is essential throughout the process.

## 5. Clinical and Therapeutic Implications of Genetic Findings

The rhGH therapy is approved in several countries for children with ISS, typically defined as a height below −2 to −2.25 SDS for age and sex, in the absence of identifiable systemic, endocrine, nutritional, or chromosomal abnormalities [5,47]. According to the 2008 consensus guidelines issued by the European Society for Paediatric Endocrinology (ESPE), GH therapy in ISS should not be administered routinely, but may be considered in carefully selected cases following comprehensive auxological and clinical evaluation [96]. This includes assessment of height velocity, mid-parental height target, bone age delay, body proportions, and exclusion of subtle skeletal dysplasias or genetic syndromes. In Italy, while no national guidelines exist specifically for ISS, current clinical practice generally adheres to ESPE recommendations. The Italian Medicines Agency (AIFA) recognizes GH therapy for a number of conditions, including but not limited GHD, SHOX haploinsufficiency, Turner syndrome, and children born small for gestational age; however, ISS is not formally included among reimbursed indications.

Clinical trials and meta-analyses suggest that long-term rhGH therapy in ISS can result in modest improvements in adult height, with average gains of 4–6 cm after 4–6 years of treatment, although interindividual variability is considerable [97,98]. Some patients achieve final heights still below the normal range, while others show minimal or no response despite adherence. Predictors of better response include first-year growth response to therapy, taller mid-parental height, delayed bone maturation, and earlier initiation of therapy [97,99]. It is noteworthy that GH dosing protocols vary internationally. Several studies have shown that lower-than-conventional doses (e.g., 0.175 mg/kg/week in Japan) are often insufficient to improve adult height in ISS [100,101,102]. These findings highlight the importance of dose optimization based on individual response and genetic context. Growth response should be reassessed after 12 months of treatment, and therapy should be discontinued in non-responders [47]. The use of long-acting rhGH in ISS is still an area of uncertainty, but recent studies provide new evidence that PEGylated rhGH may be more effective than daily rhGH in promoting catch-up growth [103]. Moreover, younger age and further distance from target height SDS have been identified as potential clinical predictors of response to treatment [104].

Identifying genetic etiologies in children with short stature transforms ISS from a descriptive label to a precise diagnosis with clear implications for prognosis, treatment, and genetic counseling. The presence of specific mutations may help predict treatment response. For example, children with *ACAN* or *NPR2* mutations often show an attenuated response to rhGH, with gains in height SDS smaller than expected [105]. Conversely, some individuals with *IGF1R* mutations may respond more favorably to IGF-1 therapy [106].

Emerging therapeutic strategies offer hope for targeted interventions beyond rhGH. CNP analogs, such as vosoritide, which is currently approved for achondroplasia [107], have shown efficacy in promoting linear growth by antagonizing FGFR3-mediated inhibition. A phase 2, randomized, controlled, multicenter study has been designed to evaluate vosoritide in children with ISS [108]. IGF-1 receptor modulators are being explored to overcome partial resistance in patients with IGF1R haploinsufficiency [109]. Additionally, epigenetic drugs targeting histone methylation or chromatin remodeling—originally developed for oncology—are being investigated for syndromic growth disorders with epigenetic underpinnings, such as Weaver or Kabuki syndromes. Although these therapies are in early stages, they represent promising tools within a personalized medicine framework for ISS [82,110].

Beyond treatment response, a molecular diagnosis may uncover associated risks requiring long-term monitoring. Children with *ACAN* mutations may develop early-onset joint pain or osteoarthritis, while variants in *COL2A1* or *FLNB* may lead to orthopedic complications. In the case of *SHOX* haploinsufficiency, there is an increased risk of scoliosis and wrist deformities, which may benefit from early orthopedic evaluation and intervention.

Genetic counseling is essential to inform families about recurrence risks and the potential implications for other relatives. Identification of an autosomal dominant mutation affects recurrence risk, prenatal counseling, and the assessment of other family members. Cascade testing can identify mildly affected or undiagnosed family members and provide reproductive guidance [111]. Furthermore, a molecular diagnosis may alleviate uncertainty for families, help avoid unnecessary diagnostic tests, and support realistic expectations about growth trajectories and final height.

## 6. Rethinking the Definition and Use of ISS

In the context of modern genomics, ISS should be viewed not as a definitive diagnosis but rather as a provisional or transitional label. This shift requires updates to clinical guidelines and a redefinition of diagnostic pathways Table 2. Pediatricians and endocrinologists must be trained to recognize subtle signs suggesting monogenic disorders and understand the relevance of integrating auxological findings with genetic and biochemical assessments [112].

Although the term ISS may still be useful as a clinical descriptor, it should prompt a more comprehensive search for etiology, also exploring new potential causes [113,114]. Rather than abandoning the term ISS, it may serve as a “clinical placeholder” used until further molecular or endocrine clarification is possible. Auxology remains a vital component of pediatric growth assessment but is no longer sufficient on its own. A more precise approach integrates growth parameters with hormonal assays and genetic testing, thereby offering the opportunity to transition from descriptive to etiological diagnoses and enable personalized management strategies.

## 7. Conclusions and Future Directions

The diagnosis of ISS is undergoing a paradigm shift. No longer solely a diagnosis of exclusion, ISS is increasingly recognized as a phenotypic endpoint of diverse genetic, epigenetic, and hormonal disruptions. Recent advances in molecular technologies—particularly NGS and genome-wide sequencing—have significantly expanded our understanding of growth biology and revealed previously unrecognized mechanisms underlying impaired stature.

Genetic factors implicated in ISS range from GH–IGF1 axis mutations to transcriptional regulators, chromatin modifiers, and epigenetic machinery, often exhibiting variable expressivity and incomplete penetrance. Importantly, many of these genes exert tissue-specific effects on growth plate chondrocytes, underscoring the need for integrating molecular biology with clinical auxology. The identification of de novo or inherited variants in children previously labeled as “idiopathic” challenges the traditional dichotomy between syndromic and non-syndromic short stature and reveals a continuum of molecular etiologies.

In this evolving context, a personalized diagnostic strategy becomes essential. Clinical red flags—such as FSS with discordant phenotypes, subtle dysmorphisms, or growth deceleration—should prompt early genetic referral. Moreover, the interpretation of genetic data requires careful phenotypic correlation and often multidisciplinary input, including pediatric endocrinologists, clinical geneticists, and molecular biologists.

Looking forward, ISS should be approached as a genomically stratifiable condition, potentially amenable to individualized follow-up, targeted therapies, and tailored counseling. This approach not only improves diagnostic precision but also reshapes patient care, moving toward a new clinical model that integrates auxology, endocrinology, and genomics. Future research must focus on functional validation of candidate genes, longitudinal natural history studies, and the ethical implications of early genetic screening.

## Figures and Tables

**Table 2:** Recommended diagnostic workflow in ISS.

Diagnostic Step	Description
Clinical and auxological evaluation	Assess height SDS, growth velocity, target height, body proportions, pubertal stage, and family history.
Biochemical screening	Perform GH stimulation tests, serum IGF-1, IGFBP-3, thyroid panel, complete blood count, and celiac screening.
Identification of red flags	Look for disproportionate stature, skeletal anomalies, advanced or delayed bone age, dysmorphic features, or poor response to rhGH.
First-line genetic testing	Analyze *SHOX* via MLPA or CGH; perform karyotype in females with signs suggestive of Turner syndrome.
Second-line genetic testing	Use targeted NGS panels including genes from GH–IGF-1 axis, ECM components, and growth plate signaling pathways.
Advanced genomic testing	Apply WES or WGS for unresolved or syndromic cases.
Pre- and post-test genetic counseling	Provide structured genetic counseling to explain findings, address VUS, and guide family implications.

Abbreviations: CGH, comparative genomic hybridization; ECM, extracellular matrix; GH, growth hormone; IGF-1, insulin-like growth factor 1; IGFBP-3, IGF binding protein-3; ISS, idiopathic short stature; MLPA, multiplex ligation-dependent probe amplification; NGS, next-generation sequencing; rhGH, recombinant human growth hormone; SDS, standard deviation scores; VUS, variants of uncertain significance; WES, whole exome sequencing; WGS, whole genome sequencing.

## Abbreviations

The following abbreviations are used in this manuscript:

CDGP	Constitutional delay of growth and puberty
CGH	Comparative genomic hybridization
cGMP	Cyclic guanosine monophosphate
CNP	C-type natriuretic peptide
ECM	Extracellular matrix
ESPE	European Society for Paediatric Endocrinology
FGFR3	Fibroblast growth factor receptor 3
FSS	Familial short stature
GH	Growth hormone
GHD	GH deficiency
GHI	GH insensitivity
GHR	GH receptor
HMGA2	High-mobility group-A2
IGF-1	Insulin-like growth factor-1
IGFBP-3	IGF binding protein-3
IGF1R	IGF-1 receptor
ISS	Idiopathic short stature
JAK	Janus kinase
MAPK	Mitogen-activated protein kinase
MLPA	Multiplex ligation-dependent probe amplification
NGS	Next-generation sequencing
NPR	Natriuretic peptide receptor
rhGH	Recombinant human growth hormone
SDS	Standard deviation score
SHOX	Short stature homeobox-containing gene
STAT	Signal transducer and activator of transcription
VUS	Variants of uncertain significance
WES	Whole exome sequencing
WGS	Whole genome sequencing
